# Cross-sectional associations between effort-reward imbalance at work and oral diseases in Japan

**DOI:** 10.7717/peerj.13792

**Published:** 2022-07-21

**Authors:** Yukihiro Sato, Eiji Yoshioka, Masanori Takekawa, Yasuaki Saijo

**Affiliations:** 1 Division of Public Health and Epidemiology, Department of Social Medicine, Asahikawa Medical University, Asahikawa, Hokkaido, Japan; 2 Department of Oral and Maxillo-Facial Surgery, Asahikawa Medical University, Asahikawa, Hokkaido, Japan

**Keywords:** Work stress, Dental caries, Periodontal disease, Tooth loss

## Abstract

**Background:**

Psychological stress is a potential risk factor for oral diseases. However, evidence for the association between work stress and oral diseases is scarce. We aimed to examine the associations of work stress, according to the effort-reward imbalance model, with dental caries, periodontal status, and tooth loss.

**Methods:**

This cross-sectional study included 184 regular employees at a medical university and 435 registrants of a web research company. Work stress was assessed using the effort-reward imbalance (ERI) ratio. Dental caries and tooth loss were assessed according to the number of decayed, filled, and missing teeth (DMFT) among the set of 28 teeth. Periodontal status was assessed using a self-administered questionnaire. For the DMFT, a linear regression model was used to estimate the unstandardised coefficients. A Poisson regression model was used to estimate the prevalence ratios (PRs) of poor periodontal status.

**Results:**

Among medical university employees and employees enrolled from among the registrants of an online research company, a one-unit increase in the ERI ratio was associated with −2.81 (95% CI [−4.70 to −0.92]; *p*-value = 0.004) and −0.84 (95% CI [−1.80 to 0.12]; *p*-value = 0.085) changes in the DMFT from adjusted linear regression models, respectively. In employees enrolled from among the registrants of an online research company, a one-unit increase in the ERI ratio was also associated with 1.55 (95% CI [1.04–2.32]; *p*-value = 0.032) of the PR for poor periodontal status based on Poisson regression models.

**Conclusion:**

ERI at work was associated with an increased risk of poor periodontal status.

## Introduction

Oral diseases are one of the global public health problems (Watt et al., 2019). The risk of developing oral diseases, such as dental caries and periodontal diseases, remains high throughout life, with no decrease observed in the incidence of dental caries at any age (Whelton, 2004; Hall-Scullin et al., 2017). The incidence of periodontal diseases rapidly increases in the mid-to-late 30s, after which it decreases due to tooth loss, but the prevalence remains high (Kassebaum et al., 2014). Dental caries frequently causes severe pain which can negatively affect work performance (Sheiham & Croog, 1981; Reisine, 1988). Periodontal diseases and dental caries can cause tooth loss (Pitts et al., 2017; Kinane, Stathopoulou & Papapanou, 2017), in turn, affecting eating, sleeping, and communication (Sheiham & Croog, 1981; Reisine, 1988). Oral diseases potentially produced a $187.61 billion productivity loss in 2015 (Righolt et al., 2018). The economic burden of oral diseases seems to worsen (Kassebaum et al., 2017). Preventing oral diseases is essential.

Psychological stress is a potential risk factor for oral diseases. It can result in immune system dysfunction, increased stress hormones, cariogenic bacterial counts, and poor oral health behaviours, which exacerbate oral conditions (Gomaa et al., 2016; Sabbah, Gomaa & Gireesh, 2018). Work stress, which is common in industrialised countries such as Japan, has become more prevalent in the workplace and can negatively impact workers’ health (Siegrist, 2016). The effort-reward imbalance model is a widely accepted model for the assessment of work stress (Siegrist, 1996; Montano, Li & Siegrist, 2016). It focuses on the failure in reciprocity in terms of high efforts spent and low rewards received. The model predicts increased risks for depression (Rugulies, Aust & Madsen, 2017), hypertension (Gilbert-Ouimet et al., 2014), coronary heart disease (Li et al., 2015), and type 2 diabetes (Kumari, Head & Marmot, 2004) when effort-reward imbalance is high. Thus, psychological stress at work might also deteriorate oral health.

Our previous systematic review revealed a lack of evidence on the association of work stress with oral diseases (Sato, Saijo & Yoshioka, 2021). Notably, most previous studies did not use the widely accepted measures for work stress assessment and did not include potential confounders, such as socioeconomic status and work-related variables (Freeman & Goss, 1993; Linden, Mullally & Freeman, 1996; Genco et al., 1999; Hayashi et al., 2001; Akhter et al., 2005; Bandar, 2009; Ramji, 2011; Mahendra et al., 2011; Islam et al., 2019). In this cross-sectional study, we aimed to examine the associations of work stress, according to the effort-reward imbalance model, with dental caries, periodontal status, and tooth loss, taking potential confounders into consideration.

## Methods

### Ethics approval and informed consent

All experiments adhere to the principles of the Declaration of Helsinki and the Ethical Guidelines for Medical and Health Research Involving Human Subjects of the Japan Ministry of Health, Labour, and Welfare. This study was reviewed and approved by the Asahikawa Medical University Research Ethics Committee (No. 18273). All participants provided web-based informed consent before responding to the online questionnaire. They responded to the questionnaire if they agreed to provide informed consent and intended to participate in this survey.

### Data sources and participants

Data were collected as previously described in Sato et al. (2022). This cross-sectional study included two source populations in Japan for covering a wider range of job types: the first source population included employees (mainly healthcare workers and office workers) at a medical university, and the second source population included registrants of a web research company (mainly administrative and managerial, professional and engineering, clerical, sales, service, and manufacturing process workers).

### Survey procedures

In the first source population, we conducted a complete survey. A self-administered questionnaire survey was conducted for all 2,006 employees of a medical university, between 19^th^ October and 20^th^ December, 2020, of which 910 responded. Of the 910, 568 were regular employees aged 20–64 years. We excluded 41 regular employees who disagreed with the use of a dental examination record. A dental examination was conducted between 7^th^ December and 18^th^ December 2020 in 184 of the 527 regular employees. The mean age (with the standard deviation) was 34.4 (10.4). The proportion of women was 56.5%.

In the second source population, we selected participants based on convenience sampling. A self-administered questionnaire survey through a web research company was conducted from 30^th^ November to 16^th^ December 2020. The inclusion criterion comprised regular employees aged 20–64 years who filled in complete information on occupational status. Consequently, 3,852 participants who met the inclusion criterion completed the questionnaire survey. Subsequently, to assess the oral conditions, the participants were required to take intraoral photographs with their mobile phones and upload them between 3^rd^ December 2020, and 12^th^ January 2021. Among the 3,852 participants, 565 uploaded the photographs. The photographs were checked, and 435 participants who provided satisfactory photographs were included; thus, 435 regular employees aged 20–64 years were included. The mean age (with the standard deviation) was 45.4 (9.9). The proportion of women was 30.6%.

### Independent variable: work stress according to the effort-reward imbalance model

Work stress was assessed using a standardised short version of the effort-reward imbalance (ERI) questionnaire in Japanese (Siegrist et al., 2008; Kurioka, Inoue & Tsutsumi, 2013). The questionnaire comprised 10 items with a four-point Likert scale: three items on the effort scale (ranging between 3 and 12) and seven items on the reward scale (ranging between 7 and 28). The effort included work pressure and immersion. Rewards included money, career opportunities, job security, and esteem. The ERI ratio was calculated using an established procedure (Siegrist, Li & Montano, 2014). A high ERI ratio indicates a failure in the reciprocity of the high efforts spent and low rewards received. The ERI ratio was used as a numerical variable, as per the manual (Siegrist, Li & Montano, 2014). In brief, the ERI ratio was calculated using the reward scale as the denominator and the effort scale as the numerator, with the weight to adjust for differences in the number of questions of each scale (Siegrist, Li & Montano, 2014).

### Dependent variable: the number of decayed, missing, filled teeth (DMFT) and self-reported periodontal status

Dental caries and tooth loss were assessed according to DMFT among the set of 28 teeth (excluding the wisdom teeth) (Petersen, Baez & World Health Organization, 2013). To independently assess dental caries and tooth loss, the numbers of decayed teeth (DT), filled teeth (FT), and missing teeth (MT) were also used as dependent variables. Although MT was defined as teeth missing due to caries in the permanent dentition, this study had no information on the cause of the lost teeth. In the first source population, DMFT was obtained through a dental examination conducted by one dentist, in accordance with standardised oral health survey methods following the World Health Organization guidelines (Petersen, Baez & World Health Organization, 2013).

In the second source population, DMFT was obtained from intraoral photographs taken with mobile phones. The participants took two intraoral photographs in line with examples provided. Two dental clinicians independently assessed each tooth’s status according to the WHO guidelines (Petersen, Baez & World Health Organization, 2013). The following diagnostic criteria were created to avoid the overestimation of DMFT while assessing intraoral photographs: “tooth was detected, but the condition is unknown” and “it is unclear whether the tooth is present or not.” Any differences between the two dental clinicians’ diagnoses were resolved by discussion. Table S1 shows the percentage of each undiagnosable tooth, wherein molars had a relatively higher percentage than incisors and premolars.

In both source populations, periodontal status was assessed using the same self-administered questionnaire. The questionnaire for screening poor periodontal status in employees consisted of four questions: smoking status, signs of periodontitis, symptoms of periodontitis, and experience of periodontal treatment (Yamamoto et al., 2009). At least three questions were asked, and the participants were defined as having poor periodontal status according to a cut-off point in the previous study (Yamamoto et al., 2009).

### Covariates

Based on a previous review, the following variables were selected as covariates (Sato, Saijo & Yoshioka, 2021): age, sex (men and women), smoking status (never, former, and current), annual household income (less than 5 million yen, 5 to 7.9 million yen, 8 to 9.9 million yen, and over 10 million yen), education (high school or lower, professional training college, junior college, technical college, and university or higher). Work characteristics included were, years of service with the current company, job type (administrative and managerial, professional and engineering, clerical, and others), occupational status (untitled and titled), and working hours per week. Job type categories were defined based on the Japan Standard Occupational Classification (Ministry of Internal Affairs & Communications, 2009). All covariates were obtained using a self-administered questionnaire.

### Statistical analysis

Two models were established using the simultaneous forced-entry method: the age-and sex-adjusted model and the fully adjusted model including age, sex, smoking status, annual household income, education, years of service with the current company, job type, occupational status, and working hours per week. In the models for periodontal status, smoking status was excluded because the periodontal status screening questionnaire included current smoking status. Linear regression analysis was employed to estimate the unstandardised coefficients of DMFT (and DT, FT, MT). The unstandardised coefficients can be interpreted as the expected changes in the DMFT. Although the distribution of DMFT (and DT, FT, MT) is right-skewed, linear regression models can provide valid estimations (Schmidt & Finan, 2018). A Poisson regression analysis with robust error variance was conducted to estimate the prevalence ratios (PRs) of poor periodontal status. PRs can be interpreted as relative risk (Zou, 2004). In the first source population, only one participant had poor periodontal status; therefore, no analysis was conducted.

The k-nearest neighbour imputation method using the R package "VIM" was employed independently for each source population (Kowarik & Templ, 2016). The k-nearest neighbour imputation method are widely accepted methods for imputing missing values. Two-tailed *p*-values of <0.05 were considered statistically significant, and 95% confidence intervals (CIs) were applied. All analyses were conducted using the R (ver. 4.1.0; R Foundation for Statistical Computing) for macOS.

## Results

The first source population included 184 regular employees of a medical university and the second source population included 435 regular employees enrolled from among the registrants of an online research company. Table 1 shows the characteristics, including the effort-reward imbalance ratio and dental status of the participants. The mean ERI ratios (with standard deviations) of the first source population and the second source population were 1.08 (0.34) and 1.14 (0.52), respectively. The median number of DMFT (with 1st and 3rd quantiles) of the first and second source populations were 6 (3, 11) and 8 (4, 12), respectively. The median numbers of DT, FT, and MT (with 1st and 3rd quantiles) of the first and second source populations were 0 (0, 0) and 0 (0, 0), 3 (0.75, 7) and 7 (3, 11), and 7 (3, 11) and 0 (0, 0), respectively. The percentages of patients with poor periodontal status among the first and the second source populations were 0.5% and 6.7%, respectively. In both source populations, more than 70% of the participants had university degrees or higher. In the first source population, over 70% were professional or engineering workers. The second source population mainly included administrative and managerial, professional and engineering, and clerical workers.

**Table 1:** The characteristics, including the effort-reward imbalance ratio and dental status of the participants stratified by the types of population.

			First source population (medical university employees)		Second source population (web research company registrants)	
Variables			(*n* = 184)		(*n* = 435)	
Effort-reward imbalance ratio		Mean, standard deviation	1.08	0.34	1.14	0.52
	Missing	*n*, %	4	2.2	0	0
Age		Median, 1st and 3rd quantile	31	26, 42	46	37, 53
Sex	Men	*n*, %	79	42.9	302	69.4
	Women	*n*, %	104	56.5	133	30.6
	Missing	*n*, %	1	0.5	0	9.0
Smoking status	Never	*n*, %	150	81.5	249	57.2
	Former	*n*, %	26	14.1	100	23.0
	Current	*n*, %	6	3.3	86	19.8
	Missing	*n*, %	2	1.1	0	0
Annual household income	<5 million yen	*n*, %	65	35.3	166	38.2
	5–7.9 million yen	*n*, %	40	21.7	159	36.6
	8–9.9 million yen	*n*, %	23	12.5	51	11.7
	≥10 million yen	*n*, %	37	20.1	49	11.3
	Missing	*n*, %	19	10.3	10	2.3
Education	High school or lower	*n*, %	6	3.3	42	9.7
	Professional training college, junior college, and technical college	*n*, %	42	22.8	53	12.2
	University or higher	*n*, %	136	73.9	339	77.9
	Missing	*n*, %	0	0.0	1	0.2
Years of service with the current company		Median, 1st and 3rd quantile	5	2, 10	13	7, 23
	Missing	*n*, %	6	3.3	0	0
Job type	Administrative and managerial	*n*, %	31	16.8	91	20.9
	Professional and engineering	*n*, %	139	75.5	110	25.3
	Clerical	*n*, %	0	0.0	145	33.3
	Others	*n*, %	14	7.6	89	20.5
Occupational status	Untitled	*n*, %	98	53.3	212	48.7
	Titled	*n*, %	85	46.2	215	49.4
	Missing	*n*, %	1	0.5	8	1.8
Working hours per week		Median, 1st and 3rd quantile	50	40, 60	45	40, 50
	Missing	*n*, %	7	3.8	0	0
Decayed, missing, and filled teeth (DMFT)		Median, 1st and 3rd quantile	6	3, 11	8	4, 12
Decayed teeth (DT)		Median, 1st and 3rd quantile	0	0, 0	0	0, 0
Filled teeth (FT)		Median, 1st and 3rd quantile	3	0.75, 7	7	3, 11
Missing teeth (MT)		Median, 1st and 3rd quantile	3	2, 3	0	0, 0
Periodontal status	None	*n*, %	178	96.7	406	93.3
	Poor	*n*, %	1	0.5	29	6.7
	Missing	*n*, %	5	2.7	0	0

Table 2 shows the association of the effort-reward imbalance ratio with the DMFT. In the first and second source population, a one-unit increase in the ERI ratio was associated with −2.81 (95% CI [−4.70 to −0.92]; *p*-value = 0.004) and −0.84 (95% CI [−1.80 to 0.12]; *p*-value = 0.085) changes in the DMFT from a fully adjusted linear regression model, respectively. Table 3 shows that a one-unit increase in the ERI ratio was associated with a 1.55 (95% CI [1.04–2.32]; *p*-value = 0.032) risk of poor periodontal status in the second source population.

**Table 2:** Associations between the effort-reward imbalance ratio and the number of decayed, missing, and filled teeth after imputation.

		First source population (employees at a medical university)					
		(*n* = 184)					
Linear regression models		Age and sex-adjusted model			Fully adjusted model		
Dependent variable	Independent variable	Unstandardised coefficient	95% CI	*p*-value	Unstandardised coefficient	95% CI	*p*-value
Decayed, missing, and filled teeth (DMFT)	Effort-reward imbalance ratio	−2.50	[–4.30 to −0.69]	0.007	−2.81	[−4.70 to −0.92]	0.004
Decayed teeth (DT)	Effort-reward imbalance ratio	0.00	[−0.26 to 0.26]	0.990	−0.06	[−0.34 to 0.21]	0.645
Filled teeth (FT)	Effort-reward imbalance ratio	−2.45	[−4.08 to −0.82]	0.003	−2.57	[−4.28 to −0.87]	0.003
Missing teeth (MT)	Effort-reward imbalance ratio	−0.05	[−0.71 to 0.62]	0.887	−0.17	[−0.88 to 0.53]	0.627

		Second source population (registrants of a web research company)					
		(*n* = 435)					
Linear regression models		Age and sex-adjusted model			Fully adjusted model		
Dependent variable	Independent variable	Unstandardised coefficient	95% CI	*p*-value	Unstandardised coefficient	95% CI	*p*-value
Decayed, missing, and filled teeth (DMFT)	Effort-reward imbalance ratio	−0.82	[−1.75 to 0.10]	0.080	−0.84	[−1.80 to 0.12]	0.085
Decayed teeth (DT)	Effort-reward imbalance ratio	0.00	[−0.10 to 0.1]	0.963	−0.03	[−0.14 to 0.08]	0.641
Filled teeth (FT)	Effort-reward imbalance ratio	−0.62	[−1.49 to −0.25]	0.163	−0.60	[−1.51 to −0.30]	0.193
Missing teeth (MT)	Effort-reward imbalance ratio	−0.21	[−0.48 to 0.07]	0.137	−0.21	[−0.49 to 0.07]	0.136

**Notes:**

The fully adjusted model included age, sex, smoking status, annual household income, education, years of service with the current company, job type, occupational status, and working hours per week.

CI, confidence interval.

**Table 3:** Associations between the effort-reward imbalance ratio and periodontal status after imputation.

		Second source population (registrants of a web research company)					
		(*n* = 435)					
Poisson regression models with a robust error variance		Age and sex-adjusted model			Fully adjusted model		
Dependent variable	Independent variable	PR	95% CI	*p*-value	PR	95% CI	*p*-value
Poor periodontal status	Effort-reward imbalance ratio	1.56	[1.06–2.29]	0.023	1.55	[1.04–2.32]	0.032

**Notes:**

The fully adjusted model included age, sex, annual household income, education, years of service with the current company, job type, occupational status, and working hours per week.

In the first source population, the number of participants with poor periodontal status was only one; therefore, no analysis was conducted.

CI, confidence interval; PR, prevalence ratio.

## Discussion

This study reports the cross-sectional associations of work stress, according to the ERI, with dental caries, poor periodontal status, and tooth loss. ERI was associated with a low number of dental caries and tooth loss, and an increased prevalence of poor periodontal status.

The current results show a negative association between work stress and the number of decayed and filled teeth (caries experienced teeth). There is only one previous study on work stress and the number of teeth with caries (Segura Marcenes & Sheiham, 1992; Sato, Saijo & Yoshioka, 2021). In a previous study, work stress was assessed using the demand-control model, and the dependent variable was DMF tooth surfaces. One unit increases in work mental demand and work control score were associated with 0.19 (95% CI [−0.91 to 1.29]) and 0.87 (95% CI [0.18–1.91]) increases in DMF teeth surfaces. This inconsistency might be due to the DMFT and DMF teeth surfaces which included caries experiences that occurred before exposure to work stress. For a more accurate understanding of the association between work stress and dental caries, the incidence of dental caries should be measured.

In the present study, work stress was negatively associated with tooth loss. There were two previous cross-sectional studies in which the dependent variables were self-reported tooth loss and having four or more lost teeth (Hayashi et al., 2001; Sato et al., 2020; Sato, Saijo & Yoshioka, 2021). Two studies have reported an increased risk of tooth loss due to work stress. Our results are inconsistent with those of the two previous studies. In this study, the number of tooth losses were few. Furthermore, as with dental caries, this inconsistency might be due to the characteristic of the dependent variable, missing teeth, which included tooth loss that occurred before exposure to work stress. Owing to these limitations, it is difficult to derive conclusions from the current results. The incidence of tooth loss should be used as an outcome in future research.

Work stress was associated with an increased risk of poor periodontal status, which is consistent with previous studies (Sato, Saijo & Yoshioka, 2021). Eight of nine previous studies reported a significant association between work stress and periodontal status, but only one study used the accepted measure for work stress and adjusted for potential confounders (Segura Marcenes & Sheiham, 1992; Sato, Saijo & Yoshioka, 2021). Our results support the previous study results.

Work stress was associated with a risk of poor periodontal status and not with dental caries and tooth loss. According to previous reviews (Tikhonova et al., 2018; Castro et al., 2020), periodontal diseases seem to be more sensitive to psychological stress than dental caries. Work stress brings about uncontrolled metabolism and impaired immune system, which can exacerbate periodontal diseases (Kinane, Stathopoulou & Papapanou, 2017). Besides, the definition of periodontal diseases is based on current inflammation activities in the supporting structures of the teeth (Chapple et al., 2018). The above features might facilitate the observation of the cross-sectional association between work stress and poor periodontal status than dental caries and tooth loss.

This study has two strengths. First, work stress was assessed using a widely accepted measure. Previous studies on work stress and oral diseases have often used nonspecific questionnaires (Sato, Saijo & Yoshioka, 2021). This study captured work stress more accurately than previous studies. Second, this study included potential confounders, such as socioeconomic status and work-related variables. Our review revealed only two studies among the 11 studies included in the systematic review considered the potential confounders sufficiently (Sato, Saijo & Yoshioka, 2021). Our study is superior to previous studies in terms of these two points. However, our study had three limitations. First, periodontal status was assessed using a self-reported questionnaire which potentially has a self-reported bias (Yamamoto et al., 2009; Beltrán-Aguilar et al., 2012). This questionnaire was not validated among young or female workers (Yamamoto et al., 2009). Furthermore, the cut-off point of this questionnaire was defined based on 7 mm or more of the clinical attachment loss, which differs from the 2018 periodontitis case definition by the European Federation of Periodontology and American Association of Periodontology with Centers for Disease Control (Botelho et al., 2020). This limitation can produce a bias. In addition, the participants with work stress may have had exaggerated responses to questions on periodontitis screening. When interpreting the periodontal status results, these limitations should be considered. In the second source population survey, DMFT was estimated using intraoral photographs with mobile phones. Molars had a relatively higher percentage of undiagnosable status than incisors and premolars, and some DT and FT were missed. Therefore, DMFT could have been underestimated in the second source population. This unvalidated method can bring about a bias. However, the results in the second source population were consistent with those in the first source population, for which DMFT was assessed on the clinical examination. Second, in the two-source population, many participants had a high socioeconomic status. More than 70% of participants had university degrees or higher. The social gradients of oral diseases have been reported (Borrell & Crawford, 2012; Schwendicke et al., 2015). Additionally, blue-collar workers were also small in number. Participants in the two-source population had better oral conditions than those in the national survey (Ministry of Health, Labour & Welfare, 2016). The results of people with low socioeconomic status and blue-collar workers might have been underestimated in this study. In addition, in the first source population, 358 participants were dropped out because they did not attend the dental examination. This loss of participants can produce a bias; however, the basic characteristics of included participants were almost similar to those of dropped out participants (Table S2). Finally, this study was cross-sectional; therefore, a temporal association was not established. Further cohort studies are required.

## Conclusions

Effort-reward imbalance at work was associated with a low number of dental caries and tooth loss, and an increased prevalence of poor periodontal status. One feature of oral diseases is causing mostly irreversible destruction of teeth and periodontal tissue. Therefore, previous studies highlight the importance of preventive care for oral diseases (Watt et al., 2019). To prevent oral diseases, occupational specialists should recognise that work stress is associated not only with mental health, cardiovascular disease, and metabolic disease, but also with periodontal status. Furthermore, support from supervisors might attenuate the negative impact of ERI on tooth loss (Sato et al., 2020). Building a supportive work environment might be also a preventive factor for oral diseases in workplaces. Moreover, periodontal diseases potentially bring about presenteeism among workers (Yoshiono et al., 2017; Zaitsu et al., 2020; Sato et al., 2022). The importance of periodontal health in the workplace should be noted. For dental caries and tooth loss, a future cohort study including the incidence of oral diseases is needed to describe the association of work stress more accurately.

## Supplemental Information

10.7717/peerj.13792/supp-1 Supplemental Information 1 Percentage of undiagnosable teeth estimated from intraoral photographs taken with mobile phones.

10.7717/peerj.13792/supp-2 Supplemental Information 2 The characteristics, including the effort-reward imbalance ratio and dental status of the participants dropped out.

10.7717/peerj.13792/supp-3 Supplemental Information 3 Information on the use of the effort-reward imbalance questionnaire.

## Additional Information and Declarations

### Data Availability

The following information was supplied regarding data availability: The data in this study are restricted from public deposition due to ethical restrictions and legal framework of Japan. The data are available upon request to ys@epid.work. Permission to access the data is determined by the Asahikawa Medical University Research Ethics Committee: https://www.asahikawa-med.ac.jp/bureau/shomu/rinri/shinsei.html.
