# Supplementary material for: Cross-sectional associations between effort-reward imbalance at work and oral diseases in Japan
Source: PeerJ. 2022 Jul 21;10:e13792. doi: 10.7717/peerj.13792 (PMC9308962; doi:10.7717/peerj.13792)
Supplement: Supplemental Information 1 [file peerj-10-13792-s001.docx]

**Supplemental Table 1:**

**Percentage of undiagnosable teeth estimated from intraoral photographs taken with mobile phones.**

| **Tooth number** | **17** | **16** | **15** | **14** | **13** | **12** | **11** | **21** | **22** | **23** | **24** | **25** | **26** | **27** |
| --- | --- | --- | --- | --- | --- | --- | --- | --- | --- | --- | --- | --- | --- | --- |
| Percentage of undiagnosable teeth | 27.8% | 4.8% | 1.6% | 1.8% | 5.7% | 1.6% | 0.5% | 0.7% | 1.1% | 3.9% | 1.6% | 2.1% | 5.1% | 28.0% |
| **Tooth number** | **47** | **46** | **45** | **44** | **43** | **42** | **41** | **31** | **32** | **33** | **34** | **35** | **36** | **37** |
| Percentage of undiagnosable teeth | 10.1% | 1.8% | 0.5% | 1.6% | 3.2% | 0.9% | 0.2% | 0.2% | 0.9% | 1.4% | 1.8% | 0.9% | 1.8% | 9.0% |
