# Supplementary material for: Cross-sectional associations between effort-reward imbalance at work and oral diseases in Japan
Source: PeerJ. 2022 Jul 21;10:e13792. doi: 10.7717/peerj.13792 (PMC9308962; doi:10.7717/peerj.13792)
Supplement: Supplemental Information 2 [file peerj-10-13792-s002.docx]

**Supplemental Table 2:**

**The characteristics, including the effort-reward imbalance ratio and dental status of the participants dropped out.**

|  |  |  | **Respondents who received a dental examination in the first source population** | | **Questionnaire respondents in the first source population** | |
| --- | --- | --- | --- | --- | --- | --- |
| **Variables** |  |  | **(*n*=184)** | | **(*n*=542)** | |
| Effort-reward imbalance ratio |  | Mean, standard deviation | 1.08 | 0.34 | 1.10 | 0.37 |
|  | Missing | n, % | 4 | 2.2 | 13 | 2.4 |
| Age |  | Median, 1st and 3rd quantile | 31 | 26, 42 | 36 | 27, 45 |
| Sex | Men | n, % | 79 | 42.9 | 210 | 40.1 |
|  | Women | n, % | 104 | 56.5 | 312 | 59.5 |
|  | Missing | n, % | 1 | 0.5 | 2 | 0.4 |
