# Supplementary material for: Cross-sectional associations between effort-reward imbalance at work and oral diseases in Japan
Source: PeerJ. 2022 Jul 21;10:e13792. doi: 10.7717/peerj.13792 (PMC9308962; doi:10.7717/peerj.13792)
Supplement: Supplemental Information 3 [file peerj-10-13792-s003.docx]

The Japanese version of the effort-reward imbalance (ERI) questionnaire is administered by Prof Akizumi Tsutsumi (https://mental.m.u-tokyo.ac.jp/jstress/ERI/index.htm).

We have been accepted to use this questionnaire.
